# The Telomeric Repeats of Human Herpesvirus 6A (HHV-6A) Are Required for Efficient Virus Integration

**DOI:** 10.1371/journal.ppat.1005666

**Published:** 2016-05-31

**Authors:** Nina Wallaschek, Anirban Sanyal, Fabian Pirzer, Annie Gravel, Yasuko Mori, Louis Flamand, Benedikt B. Kaufer

**Affiliations:** 1 Institut für Virologie, Freie Universität Berlin, Berlin, Germany; 2 Division of Infectious Disease and Immunity, CHU de Québec Research Center, Quebec City, Quebec, Canada; 3 Division of Clinical Virology, Kobe University Graduate School of Medicine, Kobe, Japan; 4 Department of Microbiology, Infectious Disease and Immunology, Faculty of Medicine, Université Laval, Quebec City, Québec, Canada; Baylor College of Medicine, UNITED STATES

## Abstract

Human herpesvirus 6A (HHV-6A) and 6B (HHV-6B) are ubiquitous betaherpesviruses that infects humans within the first years of life and establishes latency in various cell types. Both viruses can integrate their genomes into telomeres of host chromosomes in latently infected cells. The molecular mechanism of viral integration remains elusive. Intriguingly, HHV-6A, HHV-6B and several other herpesviruses harbor arrays of telomeric repeats (TMR) identical to human telomere sequences at the ends of their genomes. The HHV-6A and HHV-6B genomes harbor two TMR arrays, the perfect TMR (pTMR) and the imperfect TMR (impTMR). To determine if the TMR are involved in virus integration, we deleted both pTMR and impTMR in the HHV-6A genome. Upon reconstitution, the TMR mutant virus replicated comparable to wild type (wt) virus, indicating that the TMR are not essential for HHV-6A replication. To assess the integration properties of the recombinant viruses, we established an *in vitro* integration system that allows assessment of integration efficiency and genome maintenance in latently infected cells. Integration of HHV-6A was severely impaired in the absence of the TMR and the virus genome was lost rapidly, suggesting that integration is crucial for the maintenance of the virus genome. Individual deletion of the pTMR and impTMR revealed that the pTMR play the major role in HHV-6A integration, whereas the impTMR only make a minor contribution, allowing us to establish a model for HHV-6A integration. Taken together, our data shows that the HHV-6A TMR are dispensable for virus replication, but are crucial for integration and maintenance of the virus genome in latently infected cells.

## Author Summary

Herpesviruses are ubiquitous pathogens that persist in the host for life. Two human herpesviruses (HHV-6A and HHV-6B) can integrate their genetic material into the telomeres of host chromosomes. Integration also occurs in germ cells, resulting in individuals that harbor the virus in every single cells of their body and transmit it to their offspring, a condition that affects about 1% of the human population. We set to elucidate the integration mechanism that allows these viruses to maintain their genome in infected cells. Intriguingly, HHV-6A, HHV-6B and several other herpesviruses harbor telomere sequences at the end of their genome. Removal of these sequences in the genome of HHV-6A revealed that the viral telomeres are crucial for the integration of this human herpesvirus. In addition, we demonstrate that the telomere sequences at the right and left end of the virus genome play different roles in the integration process. Taken together, our data sheds light on the integration mechanism that allows HHV-6A to integrate into somatic cells and to enter into the germ line.

## Introduction

In 2012, the two previously described variants HHV-6A and HHV-6B were classified as separate virus species based on differences regarding their genetic and biological characteristics including variations in DNA sequences (especially in the IE region), distinct restriction patterns and specific reactivity to monoclonal antibodies [1–4]. Primary infection with HHV-6B occurs during early childhood until the age of two [5–7]. HHV-6B is the most common causative agent of the febrile illness *roseola infantum* (sixth disease) and in rare cases causes severe neurological complications such as seizures and encephalitis [8, 9]. Greater than 90% of the adult human population is seropositive for HHV-6B. The epidemiology and disease association with HHV-6A, which was initially discovered in patients with lymphoproliferative disorders [10], is much less characterized.

Upon primary infection, both HHV-6A and HHV-6B (HHV-6) establishes latency, which allows the virus to persist in the host for life. First evidence for HHV-6 integration dates back to 1993 [11], but over the years both virus species were shown to integrate virtually exclusive into the telomere region of host chromosomes in latently infected cells [12–15], while no circular episomes were detected. In addition, the virus can also integrate into germ cells, resulting in vertical transmission and individuals that harbor the integrated virus in every single cell of their body. This condition is termed inherited chromosomally integrated HHV-6 (iciHHV-6) and is present in about 1% of the human population [16–24]. The actual percentage is dependent on geographic origin and demographics of the cohort and has been shown to vary from 0.6% to 2.7% in large cohort studies. In a recent study, the relative incidence of iciHHV-6A and iciHHV-6B was determined to be 41% and 59%, respectively [24]. HHV-6 can reactivate from latently infected cells and in iciHHV-6 patients, which in both cases is associated with several diseases including encephalitis as well as infections or graft rejection following transplantation [25, 26]. While the integration is a well-established phenomenon, the underlying molecular mechanism remains completely unknown.

HHV-6 has a class A genome of about 160 kb in length and is composed of one unique segment (U) that is flanked by direct repeats (DR). The HHV-6 genome harbors two distinct arrays of telomeric repeats (TMR) at the ends of its DR region. At the right genomic terminus of the DR are the perfect TMR (pTMR), a repeat array of the hexanucleotide TTAGGG identical to the human telomere sequences. At the left end of the DR are the imperfect TMR (impTMR), telomeric repeats that are interrupted by related hexamers [27–30]. The number of TMR varies from 15 to 180 copies among clinical isolates and different laboratory strains [29]; with HHV-6B having slightly longer and more complex TMR sequences [31]. Even though TMR have been identified in the HHV-6 genome many years ago, the role of the HHV-6 TMR remained elusive. We hypothesized that the TMR could facilitate the integration of HHV-6 into the telomeres of host chromosomes by homologous recombination.

In this study, we determined the role of the TMR arrays in HHV-6A replication and integration. We generated recombinant viruses that lack all TMR or only harbor either pTMR or impTMR. Analysis of their replication properties revealed that deletion of the TMR does not affect HHV-6A replication. To determine integration efficiencies of the recombinant viruses, we established an *in vitro* integration system that allowed us to measure the integration efficiency and genome maintenance by fluorescence *in situ* hybridization (FISH) and qPCR, respectively. Using this system, we could demonstrate that the TMR are essential for efficient virus integration and that the pTMR are the key component for integration, while the impTMR only play a minor role. Our study provides the first molecular evidence on the HHV-6A integration mechanism and the viral sequence elements that facilitate its integration.

## Results

### Generation of ΔTMR mutant virus

**Fig 1:**
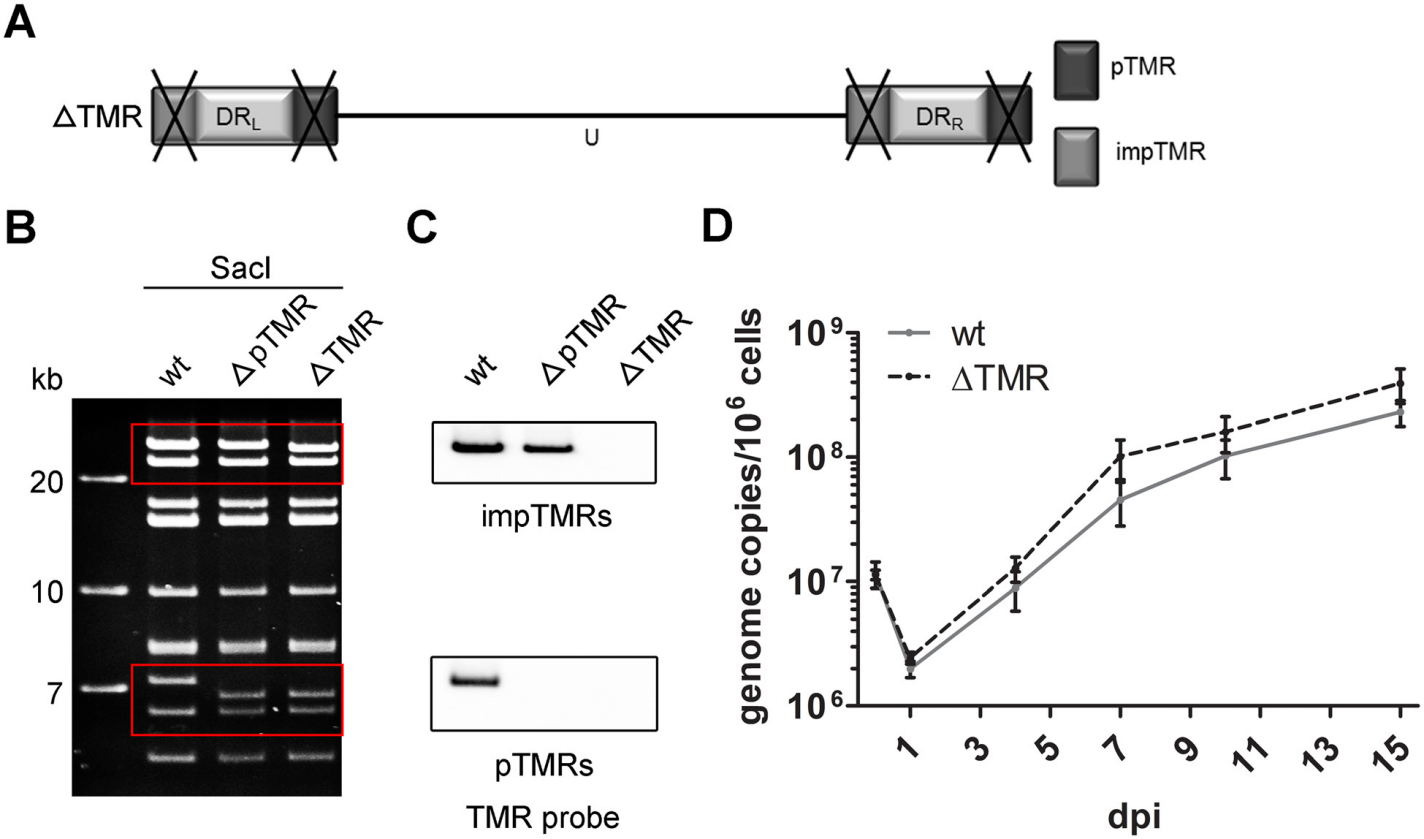
Generation and characterization of the ΔTMR mutant. **(A)** Schematic representation of the HHV-6A genome with deletion of the TMR (ΔTMR). **(B)** RFLP pattern of the wt, the ΔpTMR intermediate and the double deletion mutant ΔTMR upon digestion with *Sac*I analyzed on a 0.8% agarose gel o/n at 65 V. M = marker. Sizes of the marker fragments are indicated on the left. Red boxes highlight the fragments containing the target regions, where the expected band shifts can be observed. **(C)** Corresponding southern blot analysis detecting TMR sequences of the impTMR (upper panel) and pTMR (lower panel) after *Sac*I digestion of the indicated BAC clones using a DIG-labeled TMR probe. **(D)** Growth kinetics comparing replication properties of wt and ΔTMR mutant virus in JJHan cells. HHV-6A genome copy numbers were detected by qPCR. Copy numbers per 1x 10^6^ cells are shown as means of three independent experiments with standard errors.

To elucidate the role of the HHV-6A TMR in viral replication and integration, both TMR regions (pTMR and impTMR) were sequentially deleted in the HHV-6A BAC (ΔTMR) using *en passant* mutagenesis (Fig 1A). Mutants were analyzed by DNA sequencing, RFLP (Fig 1B) and Southern blotting, using a specific probe for the TMR (Fig 1C). Both wild type (wt) and ΔTMR mutant were reconstituted in JJHan cells by nucleofection of BAC DNA. JJHan cells are permissive for the virus and are commonly used for virus propagation. Replication kinetics in JJHan cells revealed that replication was comparable between wt and ΔTMR mutant virus (Fig 1D and S1A Fig), confirming that the TMR are dispensable for HHV-6A replication in JJHan cells.

### Deletion of the TMR severely impairs integration

**Fig 2:**
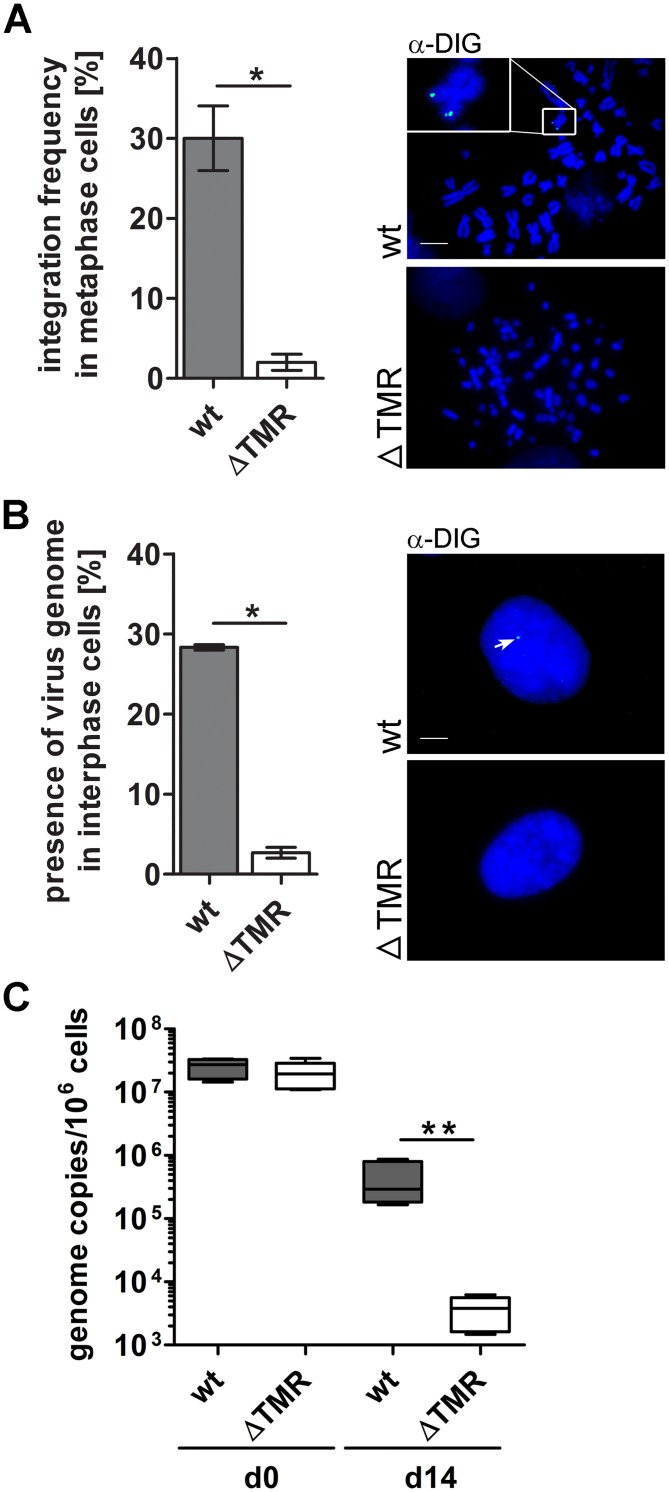
Integration efficiency and genome maintenance of the ΔTMR mutant in the U2OS integration system. **(A)** Integration frequency was quantified by determining the integration status of at least 90 metaphases. Significant differences between wt and ΔTMR (Mann-Whitney U-test, p < 0.05) are indicated with an asterisk (*). Results are shown as means of three independent experiments with standard errors. Representative metaphase images are shown on the right. Scale bar corresponds to 10μm. **(B)** 300 interphase nuclei were examined for the presence of HHV-6A. Significant differences between wt and ΔTMR (Mann-Whitney U-test, p < 0.05) are indicated with an asterisk (*). Results are shown as means of three independent experiments with standard errors. Representative interphase images are shown on the right. Scale bar corresponds to 10μm. **(C)** Maintenance of the HHV-6A genome was determined by qPCR analysis at d0 and d14 post sorting. Copy numbers per 1x 10^6^ cells are shown as means of three independent experiments with corresponding standard errors. Significant differences between wt and ΔTMR (Mann-Whitney U-test, p < 0.01) are indicated with asterisks (**).

To determine the integration efficiency of wt and ΔTMR mutant, we first established an *in vitro* integration assay using the human osteosarcoma cell line U2OS; the U2OS cells allowed the most efficient and reproducible integration of HHV-6A and HHV-6B amongst several cell lines tested (Gravel et al., submitted). U2OS cells were infected by co-seeding with JJHan cell-associated wt and ΔTMR viruses that express GFP under the control of the HCMV major immediate early promoter. JJHan cells were removed by stringent washes and GFP positive U2OS cells were sorted 36 h post infection. The pure infected cell population was cultured and used for further analyses. After 14 days, no lytic replication was detected by FISH and almost all cells lost the GFP expression, indicating that HHV-6A either established latency or the virus genome was lost. To determine if deletion of the TMR had an effect on the integration of HHV-6A, we performed metaphase FISH and could demonstrate that integration was almost abolished in the ΔTMR mutant virus compared to wt virus, which integrated in about 30% of the cells (Fig 2A). The integration observed in the U20S cells was not attributed to fusion events between JJHan and U2OS cells, as we did not observe multi-nucleated cells that would be indicative of fusion. In addition, we obtained comparable integration frequencies upon infection of U2OS cells with cell-free HHV-6A (S2 Fig). To ensure that this is not a metaphase specific effect, we analyzed interphase nuclei and observed the same phenomenon (Fig 2B). Furthermore, we generated clonal U2OS cell lines to further characterize potential integration events. Out of 92 clonal cell lines generated for the ΔTMR virus, none was positive for HHV-6A analyzed by qPCR analysis while wt lines could be readily generated. To determine if the maintenance of the virus genome was impaired in the absence of the TMR, we performed qPCR analyses at d0 and d14 post sort. Even though both viruses had similar levels at d0, genome copy numbers of the ΔTMR virus were significantly reduced compared to the wt virus at d14 (Fig 2C), suggesting that the virus genome is inefficiently maintained in the absence of the TMR. Our data demonstrate that the viral TMR play a crucial role in the integration of HHV-6A and that this process is essential for the maintenance of the virus genome.

### The pTMR are the major factor for HHV-6A integration

**Fig 3:**
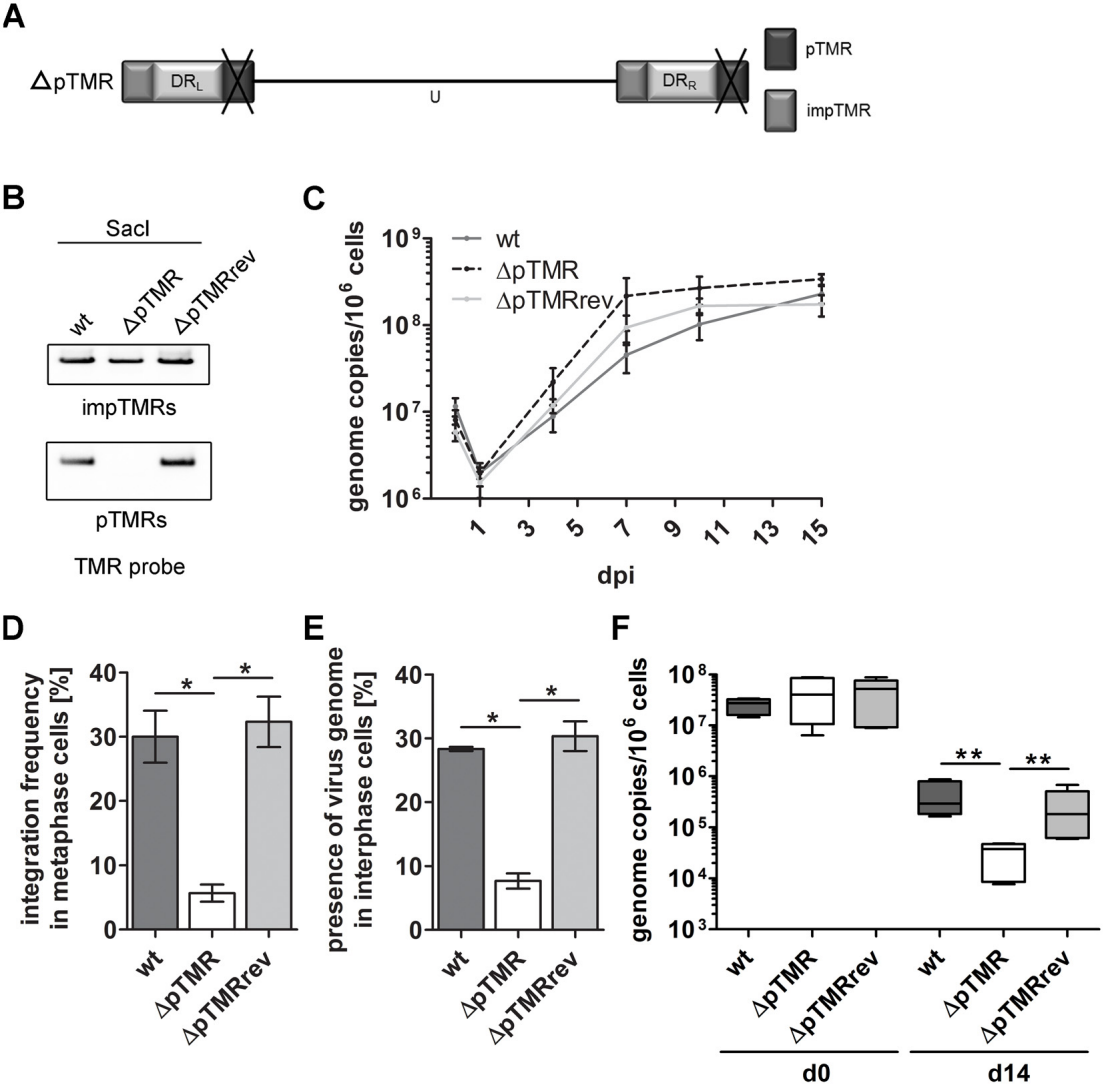
Generation, characterization and integration efficiency of the ΔpTMR mutant. **(A)** Schematic representation of the HHV-6A genome with deletion of the pTMR (ΔpTMR). **(B)** Southern blot analysis detecting TMR sequences of the impTMR (upper panel) and pTMR (lower panel) after *Sac*I digestion of the indicated BAC clones using a DIG-labeled TMR probe. **(C)** Growth kinetics comparing replication properties of wt, ΔpTMR mutant and ΔpTMRrev virus in JJHan cells. HHV-6A genome copy numbers were detected by qPCR. Copy numbers per 1x 10^6^ cells are shown as means of three independent experiments with standard errors. **(D)** Integration frequency was quantified by determining the integration status of at least 90 metaphases. Significant differences between wt and ΔpTMR, as well as ΔpTMR and ΔpTMRrev (Mann-Whitney U-test, p < 0.05) are indicated with an asterisk (*). Results are shown as means of three independent experiments with standard errors. **(E)** 300 interphase nuclei were examined for the presence of HHV-6A. Significant differences between wt and ΔpTMR, as well as ΔpTMR and ΔpTMRrev (Mann-Whitney U-test, p < 0.05) are indicated with an asterisk (*). Results are shown as means of three independent experiments with standard errors. **(F)** Maintenance of the HHV-6A genome was determined by qPCR analysis at d0 and d14 post sort. Copy numbers per 1x 10^6^ cells are shown as means of three independent experiments with corresponding standard errors. Significant differences between wt and ΔpTMR, as well as ΔpTMR and ΔpTMRrev (Mann-Whitney U-test, p < 0.01) are indicated with asterisks (**).

To investigate the distinct contribution of the pTMR in the integration process, we generated a virus that lacks the pTMR (ΔpTMR) and only contains the impTMR (Fig 3A). In addition, a revertant virus (ΔpTMRrev) was generated, in which the pTMR were restored to exclude that secondary mutations affect the integration phenotype. BAC mutants were confirmed by DNA sequencing of the target region, RFLP and Southern blotting (Fig 3B). Upon reconstitution, growth kinetics revealed that the ΔpTMR virus replicated comparable to wt and revertant virus (Fig 3C and S1B Fig).

**Fig 4:**
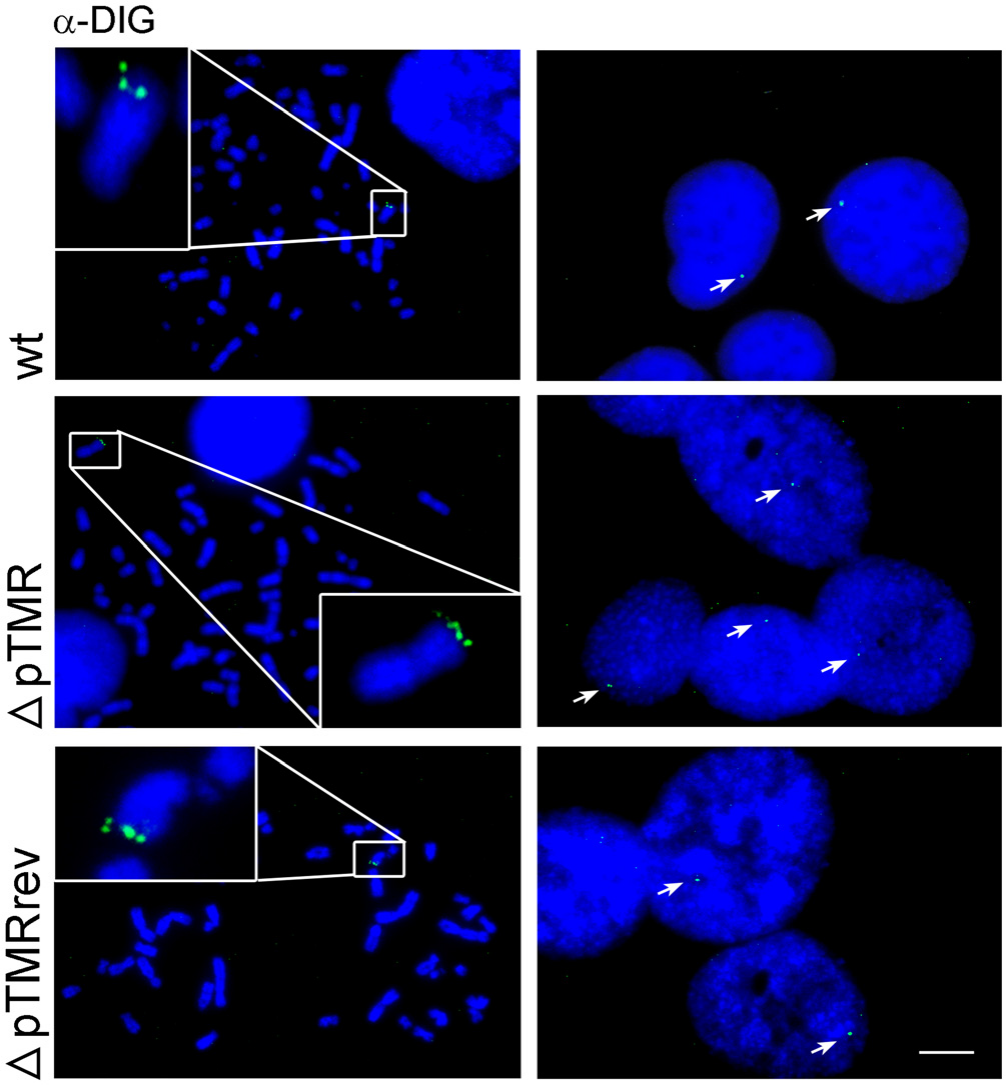
Generation of clonal U2OS cell lines containing wt, ΔpTMR and ΔpTMRrev virus. The HHV-6A genome was detected using a specific DIG-labeled probe by FISH (green) and nuclei and chromosomes were visualized using DAPI (blue). Representative images of clonal U2OS cell lines for wt (clone #9), ΔpTMR (clone #6) and ΔpTMRrev (clone #8) are shown. Metaphases are shown on the left, interphase nuclei on the right. Scale bar corresponds to 10μm.

Next, we assessed the integration efficiency of ΔpTMR using the U2OS *in vitro* integration system as described above. Integration of ΔpTMR was severely impaired, while the wt and revertant virus consistently integrated in about 30% of the metaphases (Fig 3D). Comparable results were obtained when analyzing interphase nuclei (Fig 3E). As observed for the ΔTMR mutant, the genome copies of the ΔpTMR mutant were significantly lower at d14 compared to wt and revertant (Fig 3F), indicating that the virus genome is less efficiently maintained in the absence of the pTMR. In addition, we generated clonal cell lines infected with wt, ΔpTMR or revertant virus to examine the location of the virus genome within the host chromosome. For the ΔpTMR mutant, few clonal cell lines could be established (4 out of 85). Intriguingly, ΔpTMR still integrated at the end of the host chromosomes (Fig 4), suggesting that the impTMR can facilitate integration into the telomeres with a very low efficiency. Upon stimulation with Trichostatin A (TSA), not all of the ΔpTMR clones could express early (U41) and late genes (U39), while all the wt and revertant clones did (S3 Fig). Overall, the levels of gene expression in the presence and absence of TSA were highly clone dependent; however, no increase in viral genome copy number could be observed (S4 Fig) as well as no infectious virus particles were produced with the current stimulus. In summary, our data suggests that the pTMR play an important role in integration and that the impTMR alone are not sufficient for efficient integration.

### The impTMR play a minor role in integration

**Fig 5:**
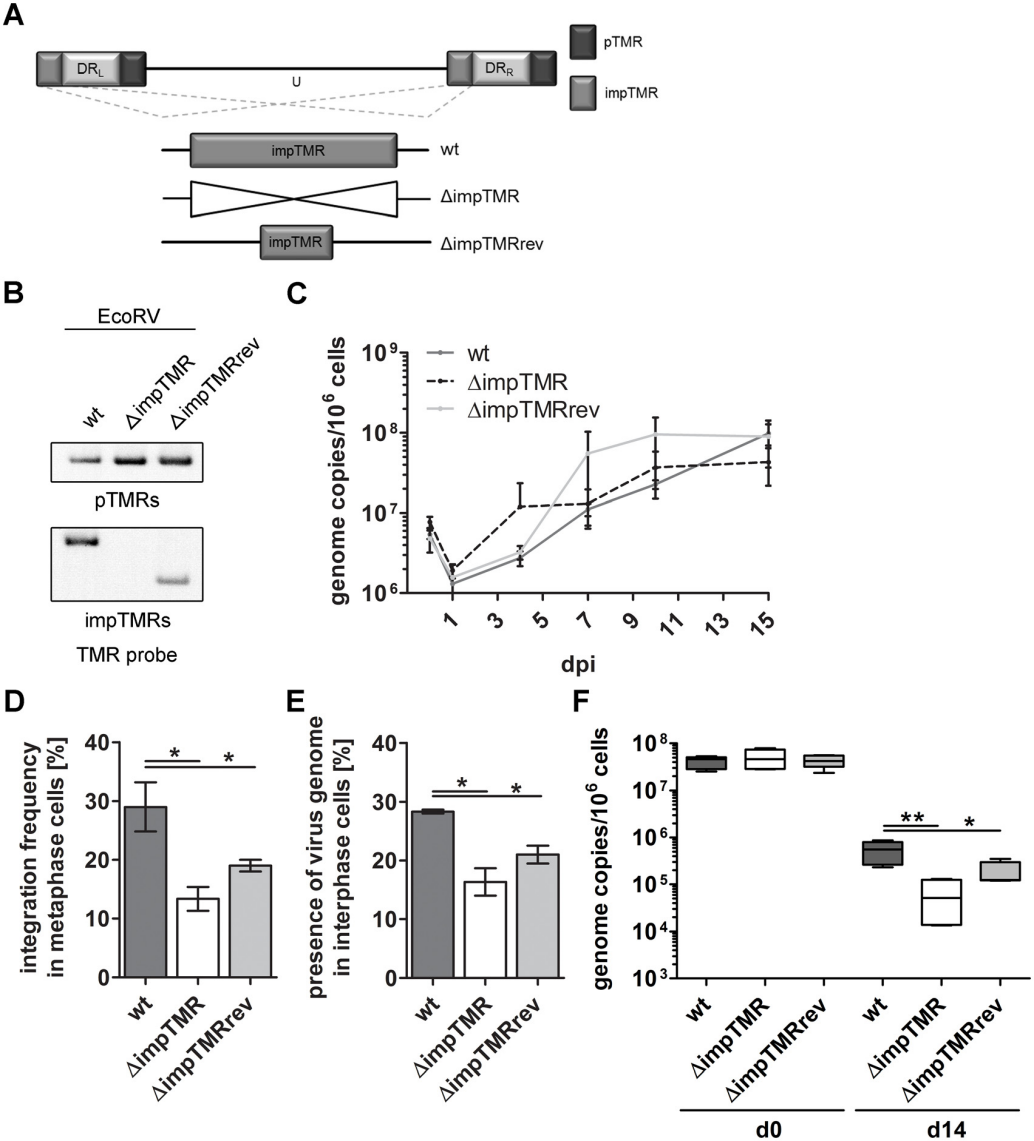
Generation, characterization and integration efficiency of the ΔimpTMR mutant. **(A)** Schematic representation of wt, ΔimpTMR and ΔimpTMRrev. **(B)** Southern blot analysis detecting TMR sequences of the impTMR (lower panel) and pTMR (upper panel) after *Eco*RV digestion of the indicated BAC clones using a DIG-labeled TMR probe. **(C)** Growth kinetics comparing replication properties of wt, ΔimpTMR mutant and ΔimpTMRrev virus in JJHan cells. HHV-6A genome copy numbers were detected by qPCR. Copy numbers per 1x 10^6^ cells are shown as means of three independent experiments with corresponding standard errors. **(D)** Integration frequency was quantified by determining the integration status of at least 90 metaphases. Significant differences between wt and ΔimpTMR, as well as wt and ΔimpTMRrev (Mann-Whitney U-test, p < 0.05) are indicated with an asterisk (*). Results are shown as means of three independent experiments with standard errors. **(E)** 300 interphase nuclei were examined for the presence of HHV-6A. Significant differences between wt and ΔimpTMR, as well as wt and ΔimpTMRrev (Mann-Whitney U-test, p < 0.05) are indicated with an asterisk (*). Results are shown as means of three independent experiments with standard errors. **(F)** Maintenance of the HHV-6A genome was determined by qPCR analysis at d0 and d14 post sorting. Copy numbers per 1x 10^6^ cells are shown as means of three independent experiments with standard errors. Significant differences between wt and ΔimpTMR, as well as wt and ΔimpTMRrev (Mann-Whitney U-test, p < 0.05 or p < 0.01) are indicated with asterisks (* or **).

To elucidate the role of the impTMR, we generated a virus that lacks the impTMR (ΔimpTMR) and only contains the pTMR (Fig 5A). While generating the recombinant viruses, we observed that the impTMR in the wt HHV-6A BAC are indeed 3 times longer (~ 2.1 kb) than reported in the corresponding NCBI reference sequence (NC_001664). To determine if the length of the impTMR has an effect on the integration, we generated a revertant virus that harbors the impTMR as reported in the NCBI Refseq (Fig 5A). Southern blotting confirmed the expected length of the impTMR region of wt and revertant virus (Fig 5B). In addition, the length of the impTMR was confirmed in the resulting viruses upon reconstitution. Growth kinetics revealed that the removal of the impTMR has no influence on virus replication (Fig 5C and S1C Fig). To determine the role of the impTMR in integration, we analyzed the integration efficiency of the recombinant viruses by FISH. In contrast to the ΔpTMR, deletion of the impTMR had only a minor effect on virus integration with two-fold reduction compared to the wt virus (Fig 5D). Intriguingly, restoration of the impTMR region to the NCBI reference sequence in the ΔimpTMRrev only partially restored the integration efficiency, indicating that the length of the impTMR region positively affects the integration efficiency. Comparable results were observed in FISH analyses of interphase cells (Fig 5E). In addition, deletion of the impTMR had only a minor effect on the maintenance of the virus genome in U2OS cells determined by qPCR (Fig 5F). Taken together, our data demonstrates that the impTMR only play a secondary role in the integration of HHV-6A.

Our data provides the first molecular evidence for the importance of the telomere repeat arrays in the integration of HHV-6A.

## Discussion

Most herpesviruses maintain their viral genome as extra-chromosomal circular episomes during latency [31]. In the case of HHV-6, Arbuckle and colleagues found no evidence for viral episomes in latently infected cells *in vitro*, but instead detected the integrated virus genome in the telomeres of host chromosomes [15]; however, the exact *in vivo* latency reservoir of HHV-6 is still poorly understood. Several groups proposed that the TMR present in the HHV-6 genome could facilitate integration [30]. This hypothesis was mainly based on sequence analyses of the integration sites in iciHHV-6 patients, as the pTMR at the right end of the viral genome were shown to be directly fused to the telomere/subtelomere sequences of the host chromosomes [15, 32–36]. However, no experimental evidence was available that the TMR are indeed involved in this process.

To address the role of the HHV-6A TMR, we generated virus mutants lacking either the impTMR (ΔimpTMR), the pTMR (ΔpTMR) or both in combination (ΔTMR) using the HHV-6A BAC. The recombinant viruses replicated comparable to wt and revertant viruses, confirming that the TMR are dispensable for HHV-6A replication and indicating that the absence of the TMR has no effect on circularization of the virus. To quantify the integration efficiency of these mutants, we established an integration assay that allowed us to address this question using a pure population of infected cells. In our FISH analyses, we observed that integration occurred in various chromosomes, judging by their size and shape, but always at the end of both chromatids of metaphase chromosomes. Episomal genomes would not specifically localize to a single chromosome and the corresponding chromatids. Using this system, we could demonstrate that HHV-6A integration is severely impaired in the absence of the TMR and that the genome is rapidly lost. Despite our efforts, we were not able to generate clonal cell lines for the ΔTMR mutant virus that harbor the HHV-6A genome, supporting our model that the TMR are central for efficient integration and maintenance of the virus genome.

Viruses that merely lack the pTMR, which display 100% identity with the human telomere sequences, also displayed significantly impaired integration capacity. This suggests that the pTMR could facilitate the first recombination event via homologous recombination with the host telomeres. Our conclusion is consistent with several studies obtaining sequence analyses in iciHHV-6 patients. The integration sites of HHV-6 *in vivo* were mapped to the pTMR of DR_R_ being fused to the telomere/subtelomere region of the human chromosomes [15, 32–36]. The impTMR alone in the ΔpTMR mutant were not sufficient for efficient viral integration, as only very few integration events occurred. The residual integration events observed for the ΔpTMR mutant virus could be explained by a recombination between the impTMR and the host telomeres. As the impTMR are not completely homologous to the host telomeres, the recombination efficiency is likely lower than for the pTMR. In patients, this less efficient integration event has already been observed in one iciHHV-6 patient, in which the impTMR could be demonstrated as the site of integration [32].

Intriguingly, deletion of the impTMR only had a minor impact on HHV-6A integration frequencies, suggesting that the impTMR are less important for the HHV-6A integration process. If the pTMR would indeed facilitate the recombination with the telomeres, the impTMR would represent the end of the host chromosome and could serve as a starting point to extend the telomeres. In this scenario, an increased length of the impTMR as we discovered in the HHV-6A wt BAC would be beneficial for the establishment of the new telomere. Upon deletion of the impTMR, the new chromosome termini (DR of HHV-6A) could be identified as a double strand break, which in turn would induce DNA damage responses. Finally, this could result in a loss of some of the cells that harbor the integrated virus genome, unless the telomeres are restored by other means.

Besides HHV-6A, already 16 other herpesviruses have been discovered that harbor TMR at the end of their genomes [31, 37]. Aside from the TMR, these viruses are very diverse, representing almost all genome classes, all *Herpesviridae* subfamilies and even the *Alloherpesviridae* that infect fish. The wide conservation of these repeats suggests that the TMR perform an important function for these herpesviruses. Our data suggest that the TMR of HHV-6A and also other viruses facilitate integration into the host telomeres, depicting a yet unappreciated mechanism for the maintenance of the virus genome in latently infected cells.

Integration does not only occur upon infection *in vitro*, but also plays an important role *in vivo*. We and other groups demonstrated that the highly oncogenic alphaherpesvirus Marek’s disease virus (MDV) can also integrate its genome into the telomeres of latently infected host chicken cells [38–40] providing thereby an optimal natural virus/host small animal model for herpesvirus integration [41]. Integration of MDV plays an important role in the pathogenesis, as it is a prerequisite for lymphoma formation in the chicken [38]. In addition, integration into host telomeres not only allows stable maintenance of the virus genome in latently infected and tumor cells but also permits efficient mobilization of the virus genome during reactivation from latency [38]. Despite recent advances, more work needs to be done to fully understand the integration mechanism that allows the maintenance of the genome of HHV-6A, MDV and potentially other viruses in latently infected cells.

## Materials and Methods

### Cells

Viruses were propagated on JJHan cells. JJHan and U2OS cells were cultured in RPMI and MEM media respectively, supplemented with 10% FBS and 1% penicillin/streptomycin. Cells were grown at 37°C, under 5% CO_2_ atmosphere. JJHan were obtained from the HHV-6 Foundation Repository (Santa Barbara, CA) and U2OS from American Type Culture Collection (ATCC, Manassas, VA)

### Generation of recombinant viruses

TMR mutant viruses were generated in pHHV-6A (wt), an infectious BAC clone of HHV-6A (strain U1102), expressing GFP under the control of the CMV IE promoter, using two-step Red-mediated mutagenesis as described previously [42–44]. Primers used for the mutagenesis are listed in (Table 1). For the TMR revertants, BAC-based transfer constructs were generated by insertion of the *I-Sce*I-*aphAI* cassette in very close proximity to the pTMR or impTMR region by Red-recombination. This BAC intermediates were used as PCR templates for mutagenesis. Recombinant BAC clones were confirmed by Southern blotting using a specific digoxigenin (DIG)-labeled probe for the TMR as described previously [38].

**Table 1:** Primers and probes for qPCR and generation of recombinant viruses.

Construct name		Sequence (5’ → 3’)
ΔpTMR	For	GGTGGCCTGGCACGGTGCCAAAGGAAACCACCGGCTAACCCATCCCCCAACGCGTAGGGATAACAGGGTAATCGATTT
	Rev	CTCCCATAGCGGCGTGCGCGCGCGCGTTGGGGGATGGGTTAGCCGGTGGTTTCCTTTGGCCAGTGTTACAACCAATTAACC
ΔpTMRrev	For	TACACACGCAGACACACAGACA
	Rev	ATACCGTCGTCCGCTCTTTC
Kana-in pTMR	For	ATCCCCCCACGCGCGCGCGCACGCCGCTATGGGAGGCGCCGTGTTTTTCACCAACACGCGCGCCGCTGCGAGACTAGGGATAACAGGGTAATCGATTT
	Rev	GTCTCGCAGCGGCGCGCGTGTTGGTGAAAAACACGGCGCCAGTGTTACAACCAATTAACC
ΔimpTMR	For	CAAATCCCCCGGGGGGGCTAAAAAAAGGGGGGGTAATAACGCTGCCCCTCTTTCACACTAGGGATAACAGGGTAATCGATTT
	Rev	GAAGCGGCAGGGGGTGATGGTGTGAAAGAGGGGCAGCGTTATTACCCCCCCTTTTTCAGTGTTACAACCAATTAACC
ΔimpTMRrev	For	GACTCCTTTTTTGTTTCGTTTTCC
	Rev	CCCAAGAGTAGCCACCAATAAT
Kana-in impTMR	For	CTTTCACACCATCACCCCCTGCCGCTTCAACTTCACCTTCTTCCTCCATCTCGCCCCGCTTGTTTCTACATAGGGATAACAGGGTAATCGATTT
	Rev	GGACAAGTGTAGAAACAAGCGGGGCGAGATGGAGGAAGCCAGTGTTACAACCAATTAACC
pTMR (seq)	For	TACACACGCAGACACACAGACA
	Rev	ATACCGTCGTCCGCTCTTTC
impTMR (seq)	For	GACTCCTTTTTTGTTTCGTTTTCC
	Rev	CCCAAGAGTAGCCACCAATAAT
TMR probe	For	TACACACGCAGACACACAGACA
	Rev	ATACCGTCGTCCGCTCTTTC
ß2M	For	CCAGCAGAGAATGGAAAGTCAA
	Rev	TCTCCATTCTTCAGTAAGTCAACTTCA
	Probe	FAM-ATGTGTCTGGGTTTCATCCATCCGACA-TAM
U86	For	TGTACATGGGCTGTAGGAGTTGA
	Rev	ACATCCTCTGCTTCCAATCTACAATC
	Probe	FAM-TTCCGAAGCAAAGCGCACCTGG-TAM
U94	For	CATGTACCAAAATGATCGATGTCA
	Rev	CCGCTTGAGCGTACCACTTT
	Probe	FAM-TGGAATAATAAAACTGCCGTCCCCACCC-TAM
U41	For	CACGATTGACAACATTTCCC
	Rev	GGGTAATGCGCATACTGAGA
	Probe	FAM-TCGCCGAACAATTTACCAGATGATTG-TAM
U39	For	CCAAGGGCCGATTATAACAC
	Rev	TCAGTCATCCGTTCAGCTTC
	Probe	FAM-TTTGCATCGACGACCCGCAT-TAM

### Virus reconstitution and growth kinetics

Recombinant viruses were reconstituted by nucleofection of JJHan cells with the HHV-6A BAC DNA as described previously [45]. Cell-free virus was generated by concentrating the supernatant of highly infected JJHan cells and stocks were frozen at -80°C. Virus stocks were titrated by analyzing the genome copies in newly infected JJHan cells by qPCR. Multi-step growth kinetics were performed as described previously [45, 46] and viral genome copies determined at 0, 1, 4, 7, 10, and 15 days post infection (dpi) by quantitative PCR (qPCR). To determine the viral gene expression level, total RNA was extracted by using the RNeasy Mini Kit (Qiagen) according to the manufacturer's protocol. The samples were then treated with DNase I (Promega). The cDNA synthesized with the high capacity cDNA reverse transcription kit (Applied Biosystems) was used for qPCR (Table 1). The viral gene expression level was normalized to the expression level of beta-2 microglobulin.

### *In vitro* integration system

To determine the integration efficiency of recombinant HHV-6A viruses, U2OS cells were infected by co-cultivation with highly infected JJHan cells for 3 h. GFP-positive cells were isolated using a FACS AriaIII cell sorter (BD, San Jose) 36 h post infection and cultured for 14 days (d). Viral genome copies were quantified by qPCR at d0 and d14 post sort relative to cellular genome copies, using primers and probes specific for HHV-6A U86 and the cellular ß2M gene (Table 1). Integration of HHV-6A was analyzed at d14 post sorting in metaphase chromosomes and interphase nuclei by fluorescent *in situ* hybridization (FISH) as described previously [38, 47, 48]. Clonal U2OS cell lines were generated upon infection of U2OS cells harboring the integrated HHV-6A genome and confirmed by qPCR and FISH.

### Statistical analyses

Statistical analyses were performed using GraphPad Prism. qPCR data of HHV-6A genome copies and integration efficiencies were analyzed using Mann-Whitney U test. Results were considered significant when p<0.05.

## Supporting Information

S1 Fig Determination of viral gene expression levels. Viral gene expression levels for the immediate early gene U86 (left panel) and the late gene U39 (right panel) were measured by qPCR and normalized to the expression level of beta-2 microglubulin. **(A)** wt and ΔTMR, **(B)** wt, ΔpTMR and ΔpTMRrev and **(C)** wt, ΔimpTMR and ΔimpTMRrev. Data of one experiment determined in triplicates is shown.
(TIF)

S2 Fig Integration frequency in U2OS cells after infection with cell-free virus. U2OS cells were infected at an MOI of 10 and the integration frequency was quantified by testing 393 clones via PCR for the presence of the HHV-6A genome. Result is shown as mean of six independent experiments with standard deviation. A representative metaphase image is shown on the right. The scale bar corresponds to 10μm.
(TIF)

S3 Fig Determination of viral gene expression level in clonal cell lines in the absence and presence of a reactivation stimulus. Various clonal U2OS cell lines (wt, ΔpTMR and ΔpTMRrev) were treated with 80 ng/ml TSA for 48 h. Viral gene expression levels for the immediate early genes U94 **(A)** and U86 **(B)**, the early gene U41 **(C)** and the late gene U39 **(D)** were measured by qPCR and normalized to the expression level of beta-2 microglobulin.
(TIF)

S4 Fig Relative genome copies of wt clonal cell lines in the absence and presence of a reactivation stimulus. Clonal cell lines were treated with TSA (80ng/μl) for 48h. HHV-6A genome copies per cell were detected by qPCR and are displayed relative to copy numbers of unstimulated cell lines. Data of one experiment determined in triplicates is shown.
(TIF)
