# Supplementary figures and images for: The Telomeric Repeats of Human Herpesvirus 6A (HHV-6A) Are Required for Efficient Virus Integration
Source: PLoS Pathog. 2016 May 31;12(5):e1005666. doi: 10.1371/journal.ppat.1005666 (PMC4887096; doi:10.1371/journal.ppat.1005666)

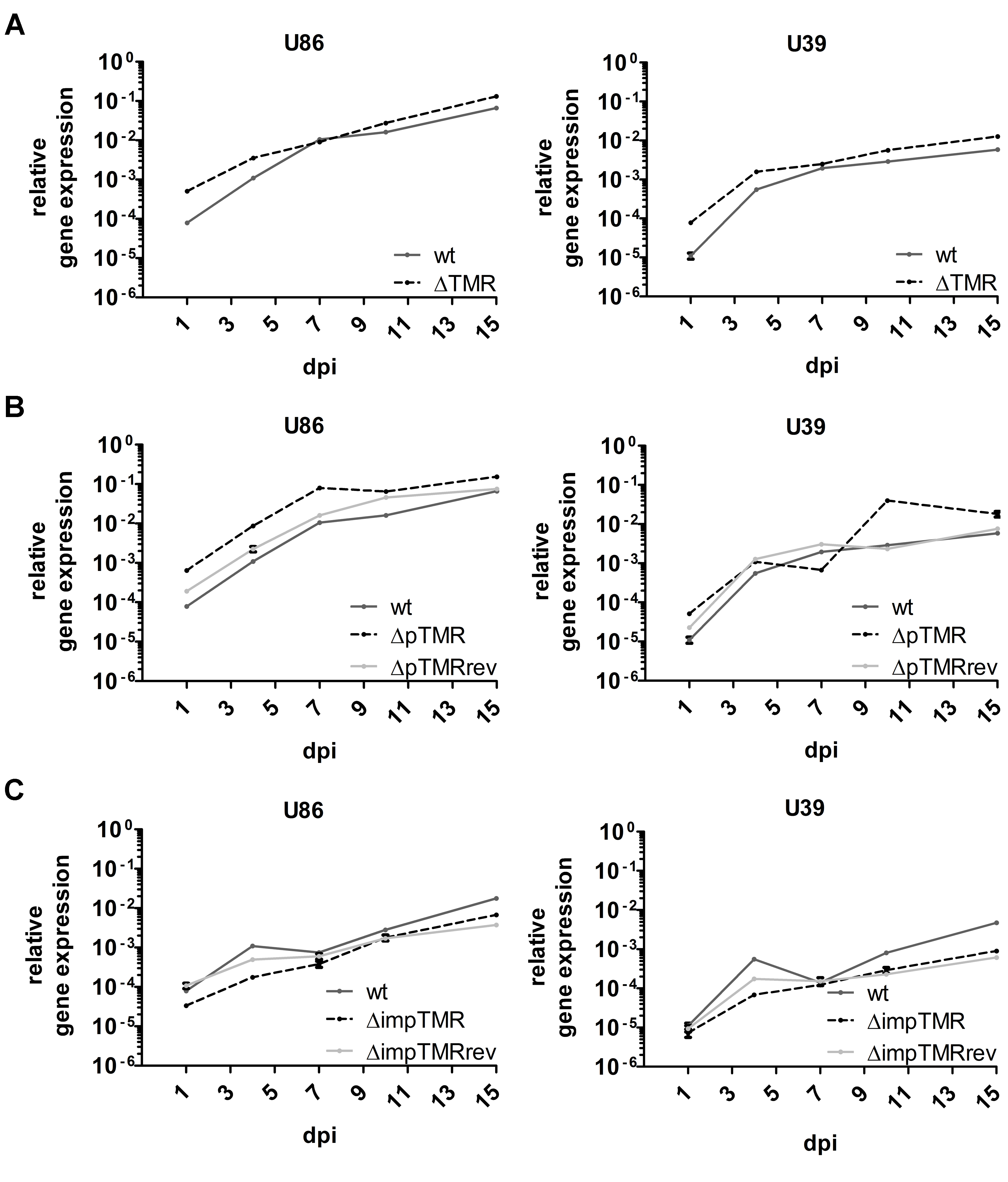

Supplement: S1 Fig — Viral gene expression levels for the immediate early gene U86 (left panel) and the late gene U39 (right panel) were measured by qPCR and normalized to the expression level of beta-2 microglubulin. (A) wt and ΔTMR, (B) wt, ΔpTMR and ΔpTMRrev and (C) wt, ΔimpTMR and ΔimpTMRrev. Data of one experiment determined in triplicates is shown. (TIF) [file ppat.1005666.s001.tif]

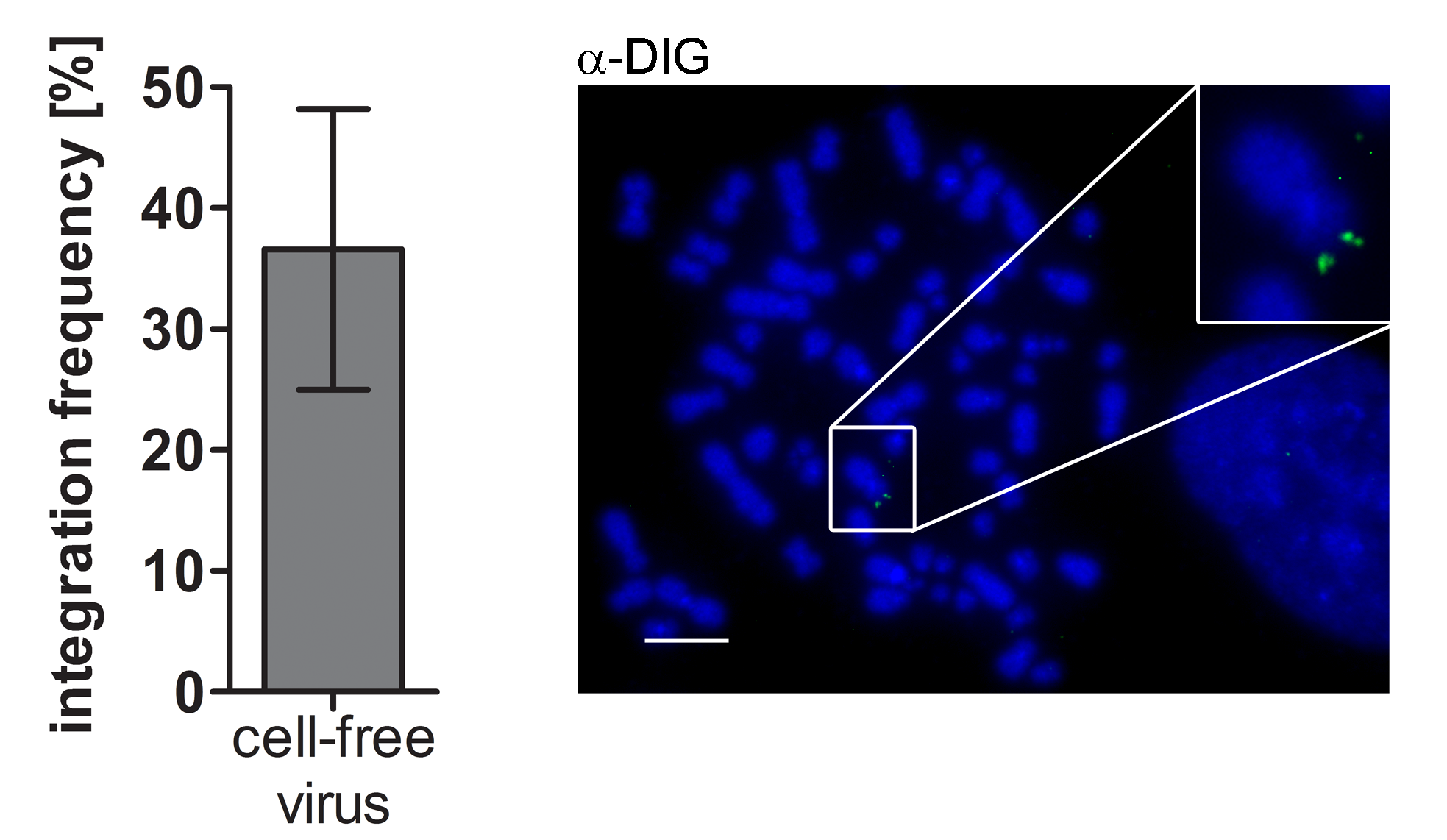

Supplement: S2 Fig — U2OS cells were infected at an MOI of 10 and the integration frequency was quantified by testing 393 clones via PCR for the presence of the HHV-6A genome. Result is shown as mean of six independent experiments with standard deviation. A representative metaphase image is shown on the right. The scale bar corresponds to 10μm. (TIF) [file ppat.1005666.s002.tif]

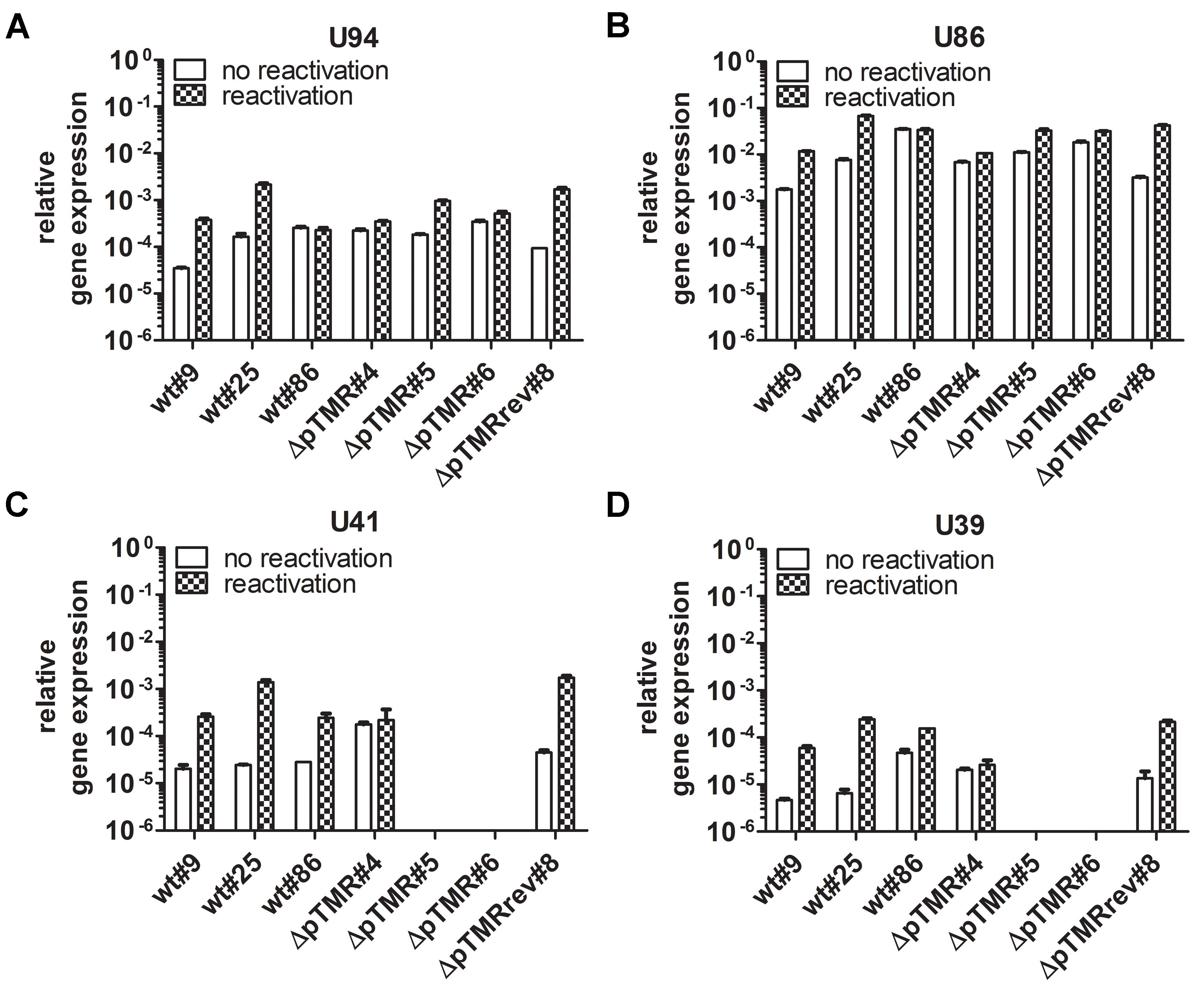

Supplement: S3 Fig — Various clonal U2OS cell lines (wt, ΔpTMR and ΔpTMRrev) were treated with 80 ng/ml TSA for 48 h. Viral gene expression levels for the immediate early genes U94 (A) and U86 (B), the early gene U41 (C) and the late gene U39 (D) were measured by qPCR and normalized to the expression level of beta-2 microglobulin. (TIF) [file ppat.1005666.s003.tif]

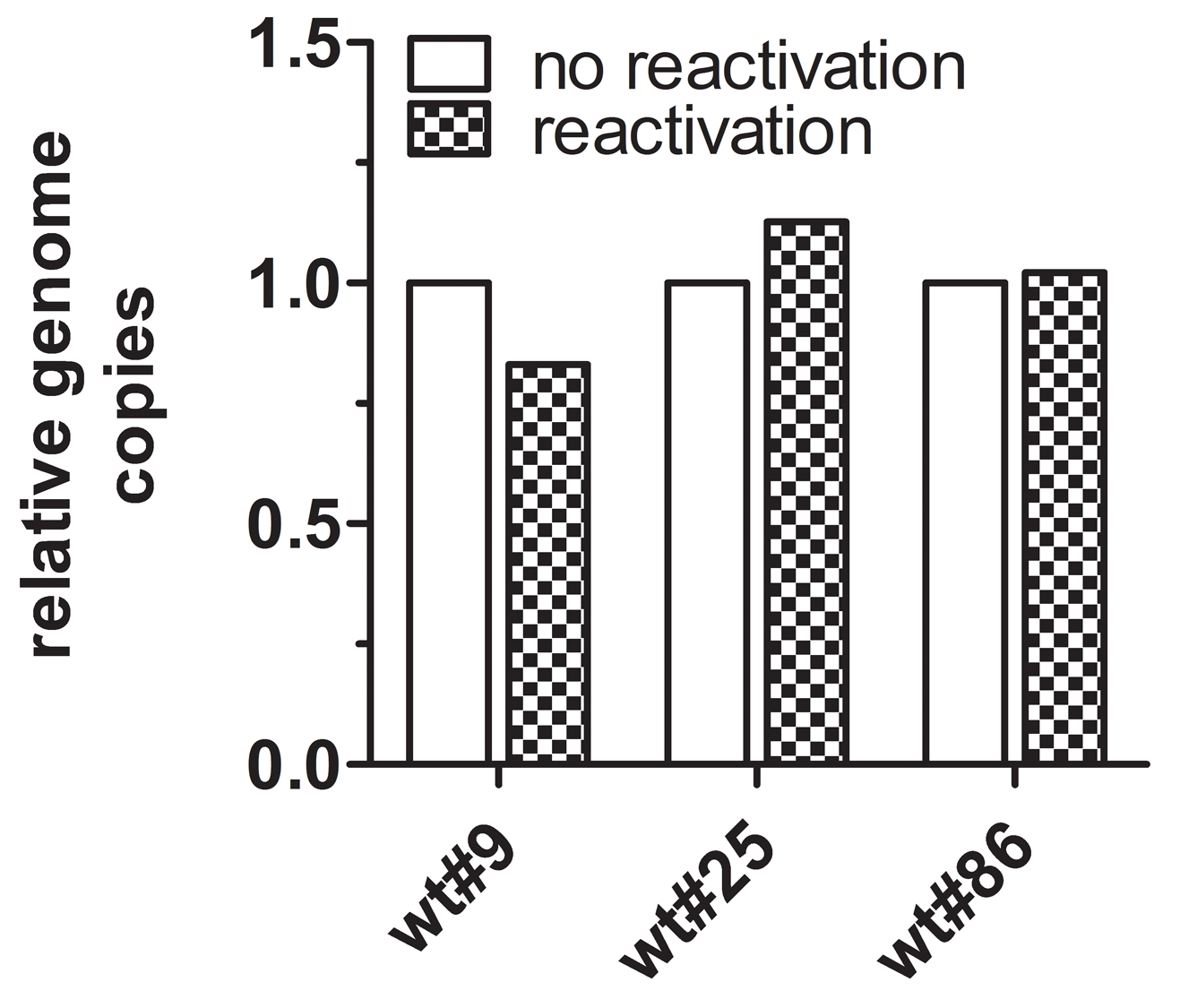

Supplement: S4 Fig — Clonal cell lines were treated with TSA (80ng/μl) for 48h. HHV-6A genome copies per cell were detected by qPCR and are displayed relative to copy numbers of unstimulated cell lines. Data of one experiment determined in triplicates is shown. (TIF) [file ppat.1005666.s004.tif]
